# Appropriateness of hip osteoarthritis management in clinical practice compared with the American Academy of Orthopaedic Surgeons (AAOS) criteria

**DOI:** 10.1007/s00264-021-05150-x

**Published:** 2021-08-17

**Authors:** Eslam Alkaramany, Abdullah Murshid, Ghalib Ahmed Alhaneedi

**Affiliations:** Orthopaedic Department, Hamad General Hospital, Hamad Medical Corporation, PO Box 3050, Doha, Qatar

**Keywords:** Appropriate use criteria, American Academy of Orthopaedic Surgeons, Hip osteoarthritis, Surgical treatment, Conservative treatment

## Abstract

**Purpose:**

The American Academy of Orthopaedic Surgeons (AAOS) developed the appropriate use criteria (AUC) for the management of hip osteoarthritis (OA) to guide surgeons in making decisions based on the best available evidence. This study aimed to assess the applicability of the AUC by comparing the actual treatment provided at our institution with the AUC recommendations.

**Methods:**

A retrospective review of 115 patients who were diagnosed and treated for hip OA at our institution between December 2017 and December 2019 was performed. Data were collected and entered into the AUC application to determine the rate of appropriateness of the provided treatment according to the AUC recommendations. Then, the actual provided treatments were compared with the AUC recommendations to determine the agreement between the two.

**Results:**

There were 115 patients, with a mean age of 50.08 years (range, 30–80 years). The most frequent patient characteristics were middle age (40–65 years) with function-limiting pain at moderate to long distances, minimal hip OA on X-ray examination, mild range of motion limitation, and presence of modifiable risk factors for negative outcomes. The overall rate of appropriateness and in agreement with the AUC recommendations was 100% for conservative treatments and 80.1% for surgical treatments.

**Conclusions:**

This study shows that the majority of the hip OA treatments provided at our institution were appropriate and in agreement with the AUC recommendations. Furthermore, the AUC can be easily accessed through a free web application using a computer or smartphone to obtain the recommended treatment for any patient with hip OA.

## Introduction

Hip osteoarthritis (OA) is a progressive, irreversible degenerative process that leads to loss of articular cartilage [1, 2]. It is considered one of the most common disabling diseases affecting the quality of life [1]. In elderly individuals above 85 years of age, there is an average 25% lifetime risk of symptomatic hip OA [1]. Different risk factors are related to the development of hip OA, including age, sex, genetics, obesity, and local joint risk factors, but the primary aetiology is still unknown [2]. Management strategies for hip OA have been developed over the years and usually start with conservative treatment followed by surgical treatment after the exhaustion of nonsurgical options [1–4].

**Fig. 1:**
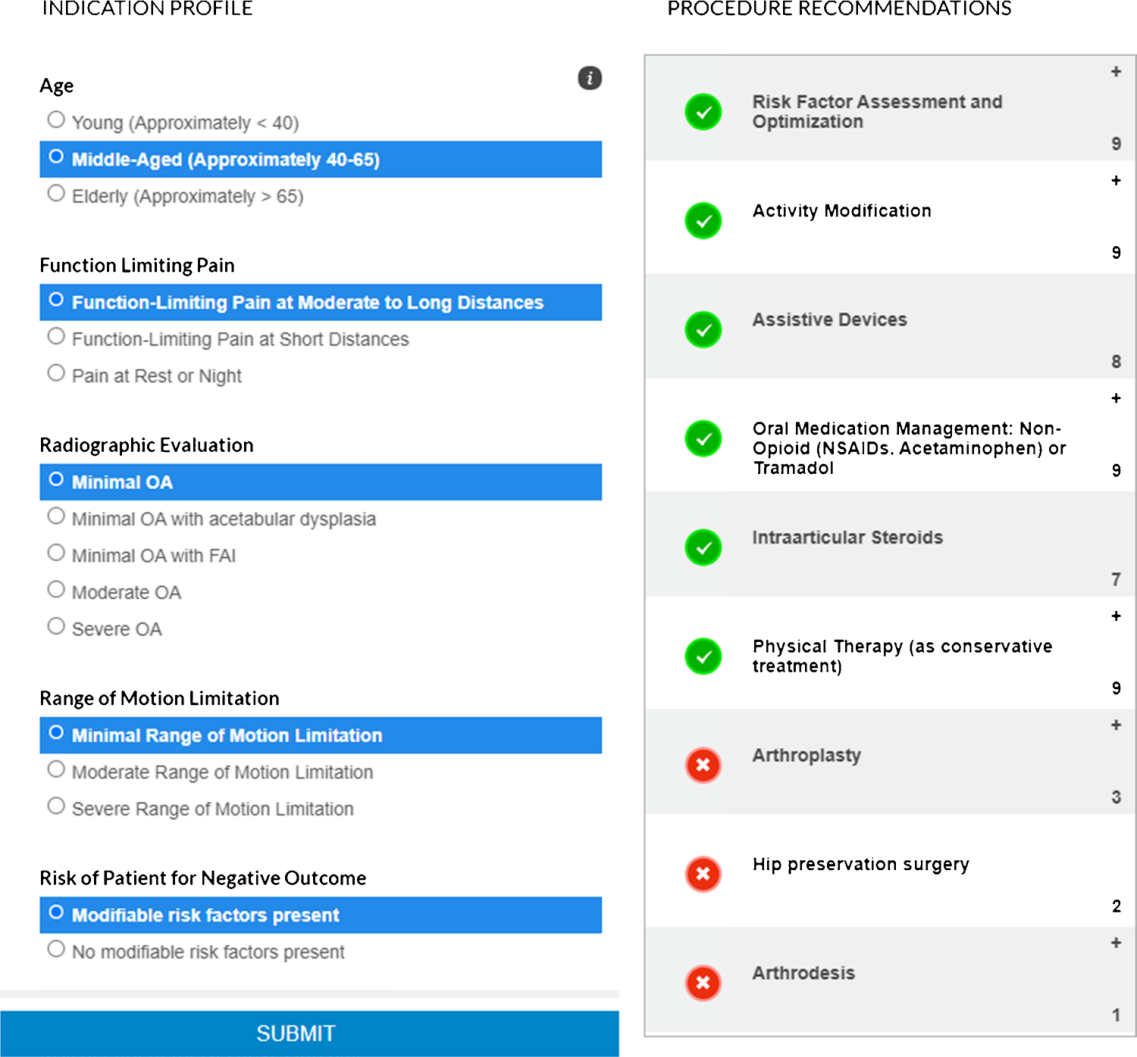
Data entry and interpretation on the AAOS appropriate use criteria free web application [5]

In December 2017, the American Academy of Orthopaedic Surgeons (AAOS) published the appropriate use criteria (AUC) based on the best available evidence to indicate the appropriateness of different healthcare services for the management of OA of the hip and made it widely available for physicians through a free web-based application (Fig. 1) [5]. These appropriate use criteria recommend non-pharmacologic and pharmacologic interventions and surgical procedures for symptomatic OA of the hip [5, 6]. The key value of the appropriate use criteria (AUC) is to guide surgeons in proper decision-making, especially in areas where gold standard randomized clinical trials are not available.

The AUC for the management of hip OA involves the assessment of five factors for each patient, including (1) age, (2) function-limiting pain, (3) radiographic evaluation, (4) range of motion limitations, and (5) risk of negative outcomes. Based on those five parameters, an appropriateness rating is generated for each of the nine suggested treatment options [6].

To the best of our knowledge, no previous studies have explored the value of the AUC as a clinical tool for the management of hip OA in clinical practice. This study aimed to assess the applicability of the AUC by comparing the actual treatment provided at our institution with the AUC recommendations.

## Methods

The Institutional Medical Research Centre approved this study (reference number, MRC-01–18-466), and the requirement for informed consent was waived. Our institution is accredited by the Joint Commission International and the Accreditation Council for Graduate Medical Education International, which requires that all treating physicians properly document all of their patient data. A retrospective review of medical charts and radiographs for patients diagnosed with hip OA at our institution between December 2017 and December 2019 was performed by two authors. Adult patients (≥ 18 years) who were diagnosed with hip OA were included. Patients with other causes of OA (inflammatory), a history of previous surgical intervention, neoplasm, neuropathy, vascular disease, or ankle or foot deformity were excluded. The two authors reviewed the files of 179 patients and ended up with a total of 115 patients with complete documentation who were eligible for inclusion.

The collected data from each patient included age, sex, function-limiting pain, radiographic evaluation according to Tonnis classification [7], range of motion limitation, risk of negative outcomes such as obesity, mental health disorders, tobacco use, or uncontrolled diabetes and the management provided.

The AUC application mandates only five patient parameters to generate an appropriateness rating for nine different treatment options for hip OA. Each treatment is rated as appropriate, may be appropriate, or rarely appropriate according to the AUC application. These nine treatments include a risk factor assessment and optimization, activity modification, assistive devices, oral medication management, intra-articular steroids, physical therapy, hip arthroplasty, hip preservation surgery, and arthrodesis.

To judge the applicability of the AUC for the management of hip OA, first, the parameters of each patient were entered into the AUC application to generate the appropriateness rating of the provided treatment for each patient. Then, the agreement between the actual surgical treatment provided at our institution and the AUC recommendations was assessed.

## Statistical analysis

Means and SDs for continuous variables and frequencies for categorical variables were used to describe the sample characteristics, patient scenarios, and treatment options. We reported the overall rate of appropriate, may be appropriate, and rarely appropriate treatments as a percentage. Then, an appropriateness rating was generated as a percentage for each of the nine treatment options.

For appropriate treatment, we reported the agreement of the actual treatment provided with the AUC recommendation as a percentage. Data analysis was performed using statistical software (IBM SPSS version 22; SPSS, Inc., Chicago, IL, USA).

No sample size calculations were performed before conducting this study because all patients who met the inclusion criteria were included. A post hoc power analysis revealed a power of greater than 80%, which showed that the sample size was adequate for analysis.

## Results

A total of 115 patients were eligible for inclusion according to the proposed criteria. The mean age was 50.08 years, and the greater number of patients were of middle age (40–65 years); 39.1% of participants were females, and 60.9% were males. Patient characteristics are summarized in (Table 1).

**Table 1:** Patients’ characteristics

Patients’ characteristics		Frequency	Percentage
**Age**	Young (approximately < 40 years)	17	14.8%
	Middle age (approximately 40–65 years)	86	74.8%
	Elderly (approximately > 65 years)	12	10.4%
**Sex**	Female	45	39.1%
	Male	70	60.9%
**Function-limiting pain**	Moderate to long distance	42	36.5%
	Short distance	65	56.5%
	At rest and night	8	7%
**Range of motion**	Minimal range of motion limitation	49	42.6%
	Moderate range of motion limitation	50	43.5%
	Severe range of motion limitation	16	13.9%
**Radiographic evaluation**	Minimal OA	35	30.4%
	Minimal OA with acetabular dysplasia	2	1.7%
	Minimal OA with FAI	8	6.9%
	Moderate OA	29	25.3%
	Severe OA	41	35.7%
**Risk factors for negative outcomes**	Modifiable risk factors present (BMI > 30/smoking/mental health/uncontrolled DM)	64	55.6%
	No modifiable risk factors present (age > 70, ethnicity)	51	44.3%

Out of the 115 patients, 36 patients received surgical management after the failure of conservative treatments, and 79 patients received 496 different conservative treatments. All 36 surgical patients underwent total hip replacement. No patients in this study underwent other surgical treatments (hip preservation surgery or arthrodesis). Operations were performed by fellowship- and non-fellowship-trained adult reconstruction surgeons. The surgical patients did not receive all conservative treatments before surgery; only five patients (13.9%) received intra-articular steroids, and approximately 16 patients (44.4%) received assistive devices. Table 2 summarizes the different conservative treatments.

**Table 2:** Percentages of conservative treatments for surgical patients

Percentages of conservative treatments received by surgical patients	
**Conservative treatments**	**Number of patients** **(*N* = 36)**
Activity modification	36 (100%)
Risk factor assessment and optimization	32 (88.9%)
Assistive devices	16 (44.4%)
Oral medication management	36 (100%)
Intra-articular steroids	5 (13.9%)
Physical therapy	36 (100%)

Regarding conservative treatments, three treatments (activity modification, oral medication management, and physical therapy) were received by all 115 (100%) patients. Other conservative treatments were distributed as follows: risk factor assessment and optimization in 100 (86.9%) patients; assistive devices in 29 (25.2%) patients; and intra-articular steroids in 22 (19.1%) patients (Table 3).

**Table 3:** AUC treatment options, rate of appropriateness, and rate of agreement

Actual treatment		Number of patients	Appropriate	May be appropriate	Rarely appropriate	Agreement with AUC recommendation	
						Yes	No
Conservative nonsurgical	Risk factor assessment and optimization	100	100 (86.9%)	-	-	100%	-
	Activity modification	115	115 (100%)	-	-	100%	-
	Assistive devices	29	29 (25.2%)	-	-	100%	-
	Oral medication management	115	115 (100%)		-	100%	-
	Intra-articular steroids	22	22 (19.1%)		-	100%	-
	Physical therapy	115	115 (100%)	-		100%	-
	**Total conservative treatments**	496	496 (100%)			100%	
Surgical	Arthroplasty	36	29 (25.2%)	7 (6%)	-	80.5%	19.5%
	Hip preservation surgery	-	-	-	-	-	-
	Arthrodesis	-	-	-	-	-	-
	**Total surgical treatments**	36	29 (25.2%)	7 (6%)	-	80.5%	19.5%
**Total treatments**		**532**	**525 (98.6%)**	**7 (1.4%)**	**-**	**98.6%**	**1.3%**

There are 270 scenarios addressed by the AUC guidelines. The most common patient characteristics in our study were middle age (40–65 years) (14.8%) with function-limiting pain at moderate to long distances, minimal hip OA on X-ray examination, mild range of motion limitation, and the presence of modifiable risk factors for negative outcomes.

After comparing the actual treatment received by the patients to the treatment recommended by the AUC for hip OA management, we found that the overall rate of treatment appropriateness and in agreement with AUC guidelines was 98.6%, although the surgeons in our study were not following the AUC guidelines during their practice. The overall rate of treatment appropriateness was 100% for conservative treatments and 80.5% for surgical treatments. Table 3 summarizes the appropriateness and agreement rates.

## Discussion

This study evaluated the appropriateness of treatment for hip OA over two years at a tertiary care centre. We found that our patient characteristics were comparable to those previously reported in the literature [2, 8]. The most valuable finding of this study was that the AUC application for hip OA management was straightforward, beneficial, and workable. Furthermore, the availability of the AUC guidelines through a free web-based application provides a great opportunity for all orthopaedic surgeons to build their practice on solid evidence-based data to improve the quality of patient care. These findings are similar to those of a study published in 2020 that assessed the usability of the AAOS AUC for the surgical treatment of knee OA and showed that the AUC can be applied easily and efficiently in clinical settings [9]. Treatments provided at our institute were found to be appropriate and in agreement with the AUC recommendations in the majority of patients, although none of the orthopaedic surgeons at our hospital had used the AUC guidelines before proceeding with the treatment plan for any patient.

It is interesting to note that the provided conservative treatments for hip OA at our institution were appropriate and in agreement with the AUC recommendations in most cases especially the activity modification, oral medication management, and physical therapy. In the literature, conservative management is considered a fundamental element in the treatment plan for hip OA. For example, Bennell reported that patient education is a core component of hip OA treatment, as it is an indispensable element in promoting adequate self-management [10]. Zhang et al. concluded that the physical therapy is the mainstay of treatment for mild and early hip OA to strengthen the hip muscles and maintain the joint mobility [11]. Last but not the least, the American College of Rheumatology recommended the use of oral medications, such as paracetamol and nonsteroid anti-inflammatory drugs (NSAIDs), for early hip OA, and opioid analgesics should be used for symptomatic hip OA with no adequate response to both non-pharmacologic and pharmacologic modalities and are either unwilling to undergo or not candidates for total joint arthroplasty [12].

We found that some conservative treatments, such as assistive devices (25.2%) and intra-articular steroids (19.1%), were less utilized than other modalities, perhaps due to underestimation of the importance of these treatments by some surgeons and a deficiency of long-term benefits.

McCabe et al. reported in a systemic review that intra-articular steroid injections may be effective for short-term pain reduction in those with hip OA, although the quality of the evidence was relatively poor [13]. Another systemic review and meta-analysis showed that intra-articular steroid injection was an effective therapy for both immediate and delayed pain reduction in hip OA patients within 12 weeks [14].

The American College of Rheumatology recommended the use of walking sticks, tap turners, canes, and other devices as adjuncts to core treatments for people with OA who have specific problems with activities of daily living [12].

For the surgically treated patients, it is concerning that the characteristics of some of our patients (approximately 19.5%) who underwent total hip arthroplasty were not in agreement with the AUC guidelines. This could be due to slight discrepancies in evidence-based surgical practice, experience, and the fact that the treating surgeons were not applying the AUC guidelines. In addition, cultural differences could play a minor role, as some patients might urge surgeons for more invasive treatments to reach the desired level of satisfaction as soon as possible.

We also noted that not all conservative treatments were attempted in surgically treated patients before surgery; for example, among operated patients, only 14% received intra-articular steroids, and approximately 44% received assistive devices. This might be due to the lack of evidence-based protocols for the treatment of hip OA at our institution. Consequently, the implementation of the AUC for hip OA management could allow surgeons to explore all available and evidence-based treatments. Currently, in the literature and according to the AUC guidelines, surgery for any patient with hip OA should be considered after the exhaustion of conservative management strategies [2, 3].

All treatment modalities for hip OA were used at our institution except for hip preservation surgery (e.g. hip arthroscopy and hip arthrodesis), probably because there were no patients in our sample with indications for such treatments and because there is a lack of strong evidence supporting the use of hip preservation surgery in hip OA. Piuzzi et al., in a systemic review, found inconclusive evidence supporting categorical indications for hip arthroscopy in the treatment of OA [15].

We observed some drawbacks to the AAOS-published AUC for the management of hip OA. The authors did not define an important parameter, i.e. the range of motion limitation. This parameter is only classified as mild, moderate, or severe according to the clinical judgement of the physician [6]. We also found that the appropriateness of combined therapy was not clearly defined in the AUC; each treatment is rated alone, and the appropriateness of combined treatments is not mentioned. The AUC recommends that surgical treatment should only be considered following dissatisfaction with appropriate non-operative treatments, yet they do not specify how many conservative treatments should be used before the conservative treatment can be considered to have failed. We believe that this determination was also left up to the surgeon’s clinical judgement. Zhang et al. concluded that the clinical guidelines advocate a combination of conservative non-drug and drug therapies for optimal hip OA management [11]. According to the AUC, the appropriateness of arthroplasty mainly depends on the patient’s age and radiological findings (OA severity), without much consideration of function-limiting pain, which is the main driver for patients to seek medical advice in most of the time. A recent paper published in 2019 by Riddle et al. explored this fact [16]. Finally, the AUC does not stress the patient’s occupation or workload as a risk factors for negative outcomes. Sulsky et al., in a systematic review published in 2012, reported that there is sufficient evidence available to identify job-related heavy lifting and standing as hazards and thus to begin developing recommendations for preventing hip OA by limiting the amount and duration of these activities [17].

Our study has several limitations, including the retrospective design, lack of a comparative group, and lack of patient outcomes to validate the AUC application in clinical practice. Incomplete documentation leading to a decrease in the sample size and cultural differences among countries might affect the appropriateness ratings. Moreover, individual radiographs may be suboptimal, which could lead to bias when interpreting the severity of OA.

## Conclusions

This study showed that the majority of the treatments for hip OA provided at our institution were appropriate and in agreement with the AUC recommendations. In addition, the AUC can be easily accessed through a free web application using a computer or mobile device to obtain the recommended treatment for any patient with hip OA.

## Data Availability

The datasets used and analysed during the current study are available from the corresponding author on reasonable request.

## References

[1] Murphy NJ, Eyles JP, Hunter DJ (2016). Hip osteoarthritis: etiopathogenesis and implications for management. Adv Ther.

[2] Lespasio MJ, Sultan AA, Piuzzi NS, Khlopas A, Husni ME, Muschler GF, Mont MA (2018). Hip osteoarthritis: a primer. Perm J.

[3] Zhang, Moskowitz RW, Nuki G (2008). OARSI recommendations for the management of hip and knee osteoarthritis, Part II: OARSI evidence-based, expert consensus guidelines. Osteoarthritis Cartilage.

[4] Pinto D, Robertson M, Hansen P, Abbott J (2012). Cost-effectiveness of nonpharmacologic, nonsurgical interventions for hip and/or knee osteoarthritis: systematic review. Value in Health.

[5] Appropriate use criteria for management of osteoarthritis of the hip. (2017) Available at: http://www.orthoguidelines.org/topic?id=1021# Accessed March 6 2020.

[6] American Academy of Orthopaedic Surgeons. Management of osteoarthritis of the hip clinical practice guideline. http://www.orthoguidelines.org/oahipguideline. Published March 3, 2017.

[7] Kovalenko B, Bremjit P, Fernando N (2018). Classifications in brief: Tönnis classification of hip osteoarthritis. Clin Orthop Relat Res.

[8] Kim C, Linsenmeyer K, Vlad S (2014). Prevalence of radiographic and symptomatic hip osteoarthritis in an urban United States community: the Framingham Osteoarthritis Study. Arthritis Rheumatology.

[9] Ahmed G, ELSweify K, Ahmed A (2020). Usability of the AAOS Appropriate Use Criteria (AUC) for the surgical management of knee osteoarthritis in clinical practice. Knee Surg Sports Traumatol Arthrosc.

[10] Bennell K (2013). Physiotherapy management of hip osteoarthritis. J Physiother.

[11] Zhang W (2005). EULAR evidence-based recommendations for the management of hip OA: report of a task force of the EULAR Standing Committee for International Clinical Studies Including Therapeutics (ESCISIT). Ann Rheum Dis.

[12] Hochberg MC, Altman RD, April KT, Benkhalti M, Guyatt G, McGowan J, Towheed T, Welch V, Wells G, Tugwell P, American College of Rheumatology (2012). American College of Rheumatology 2012 recommendations for the use of nonpharmacologic and pharmacologic therapies in osteoarthritis of the hand, hip, and knee. Arthritis Care Res (Hoboken).

[13] McCabe PS, Maricar N, Parkes MJ, Felson DT, O'Neill TW (2016). The efficacy of intra-articular steroids in hip osteoarthritis: a systematic review. Osteoarthritis Cartilage.

[14] Zhong HM, Zhao GF, Lin T, Zhang XX, Li XY, Lin JF, Zhao SQ, Pan ZJ (2020). Intra-articular steroid injection for patients with hip osteoarthritis: a systematic review and meta-analysis. Biomed Res Int.

[15] Piuzzi NS, Slullitel PA, Bertona A (2016). Hip arthroscopy in osteoarthritis: a systematic review of the literature. Hip Int..

[16] Riddle D, Perera R (2019). Appropriateness and total hip arthroplasty: determining the structure of the American Academy of Orthopaedic Surgeons System of Classification. J Rheumatol.

[17] Sulsky SI, Carlton L, Bochmann F (2012). Epidemiological evidence for work load as a risk factor for osteoarthritis of the hip: a systematic review. PLoS ONE.

